# 成都市40岁以上居民肺癌危险因素现况调查

**DOI:** 10.3779/j.issn.1009-3419.2010.11.05

**Published:** 2010-11-20

**Authors:** 勃江 陈, 为民 李, 勇 贾, 娜 郭, 丹 刘, 娴 唐, 琼 王, 俐 肖

**Affiliations:** 1 610041 成都，四川大学华西医院呼吸内科 Department of Respiratory Medicine, West China Hospital of Sichuan University, Chengdu 610041, China; 2 610041 成都，四川省成都市疾病预防控制中心 Centre for Disease Control and Prevention of Chengdu, Chengdu 610041, China; 3 610041 成都，四川省成都市武侯区疾病预防控制中心 Centre for Disease Control and Prevention of Wuhou District, Chengdu 610041, China; 4 610300 成都，四川省成都市青白江区疾病预防控制中心 Centre for Disease Control and Prevention of Qingbaijiang District, Chengdu 610300, China; 5 610045 成都，四川省成都市武侯区晋阳社区卫生服务中心 Health Center of Jinyang Community in Wuhou District, Chengdu 610045, China

**Keywords:** 现况调查, 吸烟, 肺肿瘤, 流行病学, Cross-sectional investigation, Smoking, Lung neoplasms, Epidemiology

## Abstract

**背景与目的:**

肺癌危险因素的定义尚不统一，也无一种对个体危险因素评估的公认方法。本课题组在前期研究中设计完成了《肺癌高危人群自查评分表》，并对量表的价值进行了检验。为了解成都市居民的肺癌危险因素现况，本课题组于2009年6月-2009年12月采用该量表对成都市部分居民进行问卷调查。

**方法:**

分层随机抽样抽取成都市部分40岁以上常住居民，采用《肺癌高危人群自查评分表》对其进行问卷调查。

**结果:**

按照评分标准，约21.34%居民为高危人群。总人群中男性吸烟率为48.58%，明显高于女性2.65%；5.39%的男性吸烟者在15岁之前开始吸烟；大部分个体平均每日吸烟量≤20支，吸烟持续时间20年-40年。11.34%的女性有被动吸烟，主要来源于丈夫。15.30%和5.86%的居民有油烟暴露或职业性矿物、石棉接触史；另0.77%-18.08%的个体有结缔组织疾病、肺结核、肺气肿等病史；4.91%的居民经常感到精神压抑；7.24%有肿瘤家族史。

**结论:**

成都市40岁以上居民的肺癌危险因素状况尚不乐观，但大多是可控制的吸烟、油烟暴露等不良生活习惯。应加强烟草控制、环境改善等措施，以减少肺癌的发病风险，增进人民健康。

## Abstract (en)

**Background and objective:**

In the previous studies, we have designed the Self-evaluation Scoring Questionnaire for High-risk Individuals of Lung Cancer. In order to make a better understanding of the status of risk factors of lung cancer for residents in Chengdu, we carried out the investigation from June 2009 to December 2009.

**Methods:**

With the stratified random sampling method, eligible residents were included and their risk factors of lung cancer were collected with the Self-evaluation Scoring Questionnaire for High-risk Individuals of Lung Cancer.

**Results:**

According to the criteria of the questionnaire, 21.34% of the population were at high risk of lung cancer. The smoking rate for male was 48.58%, higher than that of 2.65% for female. About 5.39% of male smokers began smoking before 15 years old. The average daily tobacco consumption in the most population was less than 20 pieces, with a duration between 20 to 40 years. However, there were 11.34% of all women suffered from passive smoking, and another 15.30% and 5.86% of residents were exposed to cooking fumes, minerals or asbestos. As for the previous illness history, 0.77%-18.08% of individuals have connective tissue diseases, pulmonary tuberculosis, emphysema and others. Finally, 4.91% of residents endured the long-term mental depression, and 7.24% had a positive family history of tumors.

**Conclusion:**

The status of risk factors for lung cancer among residents in Chengdu was not optimistic. It should be paid more attention to tobacco control and environmental improvement to improve people's health.

肺癌是目前最常见的恶性肿瘤之一。据WHO统计，全世界每年新发肺癌患者超过120万例，死亡110万例^[1]^。我国肺癌发病率和死亡率从20世纪70年代末开始，一直呈明显上升趋势，现已居城乡居民恶性肿瘤死因首位^[2]^。尽管肺癌的各种治疗方法不断改进，但患者的总体5年生存率仅约15%，且与临床分期显著负相关^[3]^。可见，肺癌的预防与早期发现、早期诊断尤为重要。为此，本课题组于2009年6月-2009年12月对成都市40岁以上居民的肺癌危险因素进行现状调查，以期为制定肺癌防治措施提供科学依据。

## 1 材料与方法

### 1.1 调查对象

根据课题组前期的样本量预算结果（7 200人），按照分层随机抽样的方法，抽取2个成都市社区——武侯区晋阳社区和青白江红阳社区，每个社区各抽取5个居委会，对其连续居住≥5年40岁以上的常住居民进行调查。

### 1.2 调查方法

以入户面访问卷调查的方式为主。问卷为本课题组前期设计的《肺癌高危人群自查评分表》^[2]^，见附表1。详细收集被调查者上述各种危险因素，并按照量表分值进行评分。

### 1.3 资料整理与分析

所有资料双人盲法录入Epidata 3.02软件数据库。经检验后导入SPSS 13.0进行统计分析。正态分布计量资料用Mean±SD表示；偏态资料用中位数表示。不同性别、年龄间的比较采用*χ*^2^检验，得分趋势采用*χ*^2^趋势检验分析。

### 1.4 质量控制

所有调查员经统一培训并经预调查考查合格后进入调查。资料录入后由专职质控员随机抽取10%对比，并电话回访被调查者，符合率≥99%为合格。

## 2 结果

### 2.1 一般情况

共调查居民7 543名。将所有资料导入SPSS 13.0，剔除明显录入缺失者287名；质控员再随机抽取774例资料，对双人盲法录入结果进行对比，2例调查对象的吸烟情况两次录入结果不一致；电话回访772例居民，3例结缔组织疾病史、1例精神压抑的回访结果与原调查结果矛盾；其他768例调查对象资料吻合，符合率99.2%（768/774）。调查获得有效问卷7 250份，有效率96.12%（7 250/7 543）。其中男性3 477名，占47.96%；女性3 773，占52.04%。年龄范围为40岁-95岁，其中40岁-60岁有4 409名（60.81%）；61岁-80岁有2 682名（36.99%）；>80岁有159名（2.20%）。量表得分最高分383分，最低分0分。在量表设计的前期调查和检验中，我们通过*meta*分析等循证医学方法筛选出15个与肺癌发生有明确统计学和临床医学意义的危险因素，并由“相对危险度（relative risk, RR）或比值比（odds ratio, OR）×人群暴露率”的方法计算各危险因素的危险分数；为便于使用，将危险分数按照等比扩大的方式转换成危险赋值，设计完成《肺癌高危人群自查评分表》。量表中的危险赋值反映了各因素在肺癌发生中的作用大小。根据相关统计学原理，各项得分之和即代表个体肺癌发病的综合风险。在预调查之后，我们纳入了312例肺癌患者和1 849例正常对照个体的资料，通过受试者工作特征曲线（receiver operating characteristic curve, ROC）确定高危与非高危人群的得分阈值为116分^[2]^。按照此标准，本次调查中有1 547名为高危人群，占21.34%，呈正偏态分布，中位分数55分，四分位数间距为77分（Q_U_-Q_L_=103-26=77）。

### 2.2 不同性别、年龄个体得分分布

按照不同性别、年龄分层，个体的量表得分情况见表 1。可见，不同年龄段，男性高危个体的比例均较女性高（*P* < 0.05）。随着年龄的增加，总人群中高危个体所占的比例呈逐渐上升趋势（*χ*^2^_趋势_=28.037，*P* < 0.001）；女性亦然（*χ*^2^_趋势_=141.76，*P* < 0.001）；而男性此趋势不明显（*χ*^2^_趋势_= 2.756，*P*=0.097）。

**1:** 不同性别、年龄个体量表评分 Sores for male and female with different ages

Age (years)	Male					Female				*X*^2^	*P*
	Total	≥116	< 116	Proportion of risk individuals (%)		Total	≥116	< 116	Proportion of risk individuals (%)		
40-60	2 019	800	1 219	39.62		2 390	59	2 331	2.47	963.1	< 0.001
61-80	1 369	488	881	35.65		1 313	149	1 164	11.35	218.5	< 0.001
> 80	89	38	51	42.70		70	13	57	18.57	10.5	0.001
Total	3 477	1 326	2 151	38.14		3 773	221	3 552	5.86	1 123	< 0.001

1 547例高危人群中85.71%（1 326名）为男性。从年龄分布看，以40岁-60岁为主，共860名，占55.60%。1 349例高危个体有现在或既往吸烟史，占87.20%，其中95例在15岁以前吸烟；963例吸烟持续时间为20年-40年，223例吸烟持续时间>40年。在198例不吸烟的高危个体中，以油烟暴露、精神压抑、女性被动吸烟、慢性支气管炎病史和结缔组织疾病史为常见危险因素，阳性率分别为77.27%（153/198）、76.26%（151/198）、54.55%（108/198）、46.46%（92/198）和42.42%（84/198）。41.92%（83/198）同时具有油烟暴露和女性被动吸烟两项危险因素；31.82%（63/198）有油烟暴露和和慢性支气管炎病史；21.21%（42/198）有慢性支气管炎和结缔组织疾病史。13.64%（27/198）有油烟暴露、女性被动吸烟和慢性支气管炎三项危险因素。得分最高者同时具有油烟暴露、精神压抑、女性被动吸烟、慢性支气管炎和结缔组织疾病五个危险因素。

### 2.3 不同性别、年龄个体吸烟情况

#### 2.3.1 吸烟率

男性吸烟者1 689人，吸烟率为48.58%；女性吸烟者100人，吸烟率为2.65%，男性吸烟率明显高于女性（*χ*^2^=2 053, *P* < 0.001）；且各年龄段男性吸烟率也显著高于女性（*P* < 0.05），见表 2。

**2:** 不同性别、年龄居民吸烟率 Ratios of smoking for male and female with different ages

Age (years)	Male				Female			*X*^2^	*P*
	Total	Smoking	Non-smoking		Total	Smoking	Non-smoking		
40-60	2 019	1 135	884		2 390	55	2 335	1 614	< 0.001
61-80	1 369	515	854		1 313	39	1 274	490.9	< 0.001
> 80	89	39	50		70	6	64	23.99	< 0.001
Total	3 477	1 689	1 788		3 773	100	3 673	2053	< 0.001

#### 2.3.2 吸烟起始年龄

有5.39%的男性吸烟者在15岁之前开始吸烟，女性则为4.00%，但两者差异无统计学意义（*P*=0.548）。除>80岁组无法进行*χ*^2^检验外，其余各年龄组男女性幼年吸烟率亦无差异（*P*>0.05），见表 3。

**3:** 不同性别、年龄居民吸烟起始时间 Initial time of smoking for male and female with different ages

Age (years)	Male				Female			*X*^2^	*P*
	≤15 years old	> 15 years old	Proportion of smokers stared smoking ≤15 years old (%)		≤15 years old	> 15 years old	Proportion of smokers stared smoking ≤15 years old (%)		
40-60	62	1 073	5.46		0	55	0.00	2.160	0.142
61-80	29	486	5.63		4	35	10.26	0.004	0.949
> 80	0	39	0.00		0	6	0.00	-	-
Total	91	1 598	5.39		4	96	4.00	0.362	0.548

#### 2.3.3 平均每日吸烟量

从表 4可以看出，男女性各年龄段均以每日吸烟量≤20支为主，每日吸烟量≤20支的男性占男性吸烟者的85.73%，女性占95.00%；而大量吸烟者，尤其是每日吸烟量>40支者较少，男女性所占比例分别为0.53%和0。

**4:** 不同性别、年龄居民平均每日吸烟量 Average daily tobacco consumption for male and female with different ages

Age (years)	Male					Female			
	1-10 cigarettes per day	11-20 cigarettes per day	21-40 cigarettes per day	> 40 cigarettes per day		1-10 cigarettes per day	11-20 cigarettes per day	21-40 cigarettes per day	> 40 cigarettes per day
40-60	498	493	142	2		33	19	3	0
61-80	225	197	86	7		26	12	1	0
> 80	21	14	4	0		2	3	1	0
Total	744	704	232	9		61	34	5	0

#### 2.3.4 吸烟持续时间

表 5显示居民吸烟持续时间多为20年-40年，吸烟持续时间多为20年-40年的男性占男性吸烟者的54.88%，女性则为47.00%。

**5:** 不同性别、年龄吸烟持续时间 Smoking duration for male and female with different ages

Age (years)	Male				Female		
	< 20 (years)	20-40 (years)	> 40 (years)		< 20 (years)	20-40 (years)	> 40 (years)
40-60	436	659	40		29	26	0
61-80	108	253	154		13	20	6
> 80	5	15	19		1	1	4
Total	549	927	213		43	47	10

### 2.4 女性被动吸烟

3 773例女性中，612例有被动吸烟。量表评分要求认为当被动吸烟≥200年支时，将明显增加肺癌的发病风险^[2]^。428例女性达到此标准，占全部女性的11.34%；其中304例来源于丈夫，占71.03%；其余来源于工作场所或公共场所。

### 2.5 其它环境因素暴露

1 109例（15.30%）长期油烟暴露史，921例为女性，占83.05%。425例（5.86%）曾从事矿工、锅炉工、电焊工等10年以上；21例因工作原因接触石棉，其中1例长达20年，另2例同时合并有5年-6年矿工工龄。

### 2.6 既往疾病

289例（3.99%）居民有结缔组织疾病，其中207例（71.63%）为类风湿性关节炎，其它包括干燥综合征、血管炎、系统性红斑狼疮、强直性脊柱炎等。仅96例患者（33.22%）坚持按医嘱正规治疗和随访，其他患者多自行对症处理。在肺结核、慢性支气管炎、肺气肿3种常见肺部疾病中，有明确肺结核史者56例，占总人数的0.77%，48例表示正规治愈，无调查对象诉近期有咳嗽、咯血、潮热、盗汗等典型结核复发表现。131例（18.08%）居民有长期咳嗽、咳痰，每年持续3个月，连续2年以上；其中84例（1.16%）既往诊断肺气肿。本次调查尚未发现器官移植患者。

### 2.7 精神压抑

356例（4.91%）因生活、工作等原因长期精神压抑。

### 2.8 肿瘤家族史

525例（7.24%）居民一、二级亲属中有肿瘤家族史，以消化道肿瘤最为常见；141例（1.94%）居民有肺癌家族史。其中24例（0.33%）二者兼有。

## 3 讨论

尽管肺癌的发病机制尚不完全清楚，但现已证实，肺癌是一种与吸烟、环境污染、遗传等多种原因有关的多因素疾病^[4]^。在各种刺激因子的共同作用下，机体组织细胞生物学行为发生改变，最终导致肺癌的形成。目前肺癌的治疗效果相对有限，让人类远离肺癌威胁的关键在于进行切实有效的预防。为此，本课题组在成都市开展了一次大规模的肺癌危险因素现况调查，旨在为制定防治措施奠定科学基础。

### 3.1 调查人群的选择

此次调查以课题组前期设计的《肺癌高危人群自查评分表》为依托，详细收集了7 000多名居民的15个方面的肺癌危险因素。该调查表在前期的预调查中已证实了具有较高的使用价值^[2]^，且调查对象来源于成都市的分层随机抽样结果，既有城市中心辖区（武侯区晋阳社区），又包括城郊农村居民（青白江红阳社区），样本量亦经过严格估算得出，因而认为此次调查具有较高的可靠程度。

一般来说，恶性肿瘤的发病率和发病危险随年龄的增长而逐渐增大。国外研究^[5, 6]^指出肺癌的高发年龄为50岁-80岁。近年来，其年轻化趋势逐渐明显。但美国癌症协会（American Cancer Society, ACS）2010年公布的不同年龄段癌症构成比显示^[7]^， < 40岁的个体最常见的肿瘤为白血病，而肺癌仍主要见于40岁以上群体。我国学者隋大鸣等^[8]^分析了1 003例肺癌患者资料，也提示 < 40岁的“青年肺癌”患者仅为48例，不到总病例数的5%。段玉忠等^[9]^纳入的1 345例肺癌病例分析结果也与之基本一致。从发病机制分析，肺癌的发生与不良环境刺激密切相关，且存在累积效应，因此多认为肺癌的发病率从40岁以后迅速增加。有数据显示35岁肺癌发病率约为3.6/10万，是肺癌总体发病率35/10万的1/10。本课题组在前期量表设计的研究中，亦发现 < 40岁不会显著增加肺癌的发病危险，其危险赋值为0分^[2]^。为此，在本次调查中，为达到最佳成本-效益比，选择40岁以上个体为调查对象。从这7 250例居民的量表得分分布来看，大部分个体得分在116分以下，仅21%左右的个体为高危人群，呈正偏态分布，符合一般统计学规律。

### 3.2 各危险因素与肺癌发生风险的关系

目前，吸烟已是公认的肺癌首要危险因素，90%的肺癌与吸烟有关^[10]^。烟草在燃烧的过程中会生成4 000余种化学物质，绝大部分对人体有害，包括尼古丁（烟碱）、一氧化碳和烟焦油等。烟焦油主要由多环芳烃和亚硝胺组成，是致肺癌的元凶，尤其是对既往已有肺部结核、慢性支气管炎、肺气肿等病变者而言，这种刺激可能更加有害。日本的一项系统评价^[11]^显示，男性吸烟者发生肺癌的危险性是不吸烟者的3.54倍（RR=4.39, 95%CI: 3.00-4.17），差异有统计学意义。一般认为吸烟与肺癌的发生存在正向剂量-效应关系。吸烟10包年者RR为1.73，而40包年以上则上升至8.41^[12]^。此外，近年来，吸烟起始年龄与肺癌的关系也受到越来越多的关注。一项历时14年、观察了681例肺癌患者的研究^[13]^指出：男性17岁之前开始吸烟者，肺癌危险性增加48%（RR=1.48, 95%CI: 1.11-1.96），而女性则更高（RR=8.07, 95%CI: 2.34-27.85）。有学者^[2]^认为，肺癌的形成不仅取决于致癌物质的强度，在更大程度上还取决于致癌物质与机体内环境中抗癌物质的相互作用。年龄越小，机体免疫功能越不完善，对致癌物质的清除功能越差。这可能是吸烟起始年龄越小，危险性越大的原因之一。本研究以15岁作为吸烟起始年龄的分界点，与上述研究相近。

本次调查的男性吸烟率为48.58%，女性为2.65%。虽然较2002年的全国调查数据（66.0%和3.08%）低，但同时也提示控烟工作尚任重而道远。大部分吸烟者烟龄在20年-40年之间，且以每日吸烟量≤20支为主。如何对这部分人群进行有效的烟草控制，将是后期社区卫生工作的重心之一。目前的控烟手段主要包括健康教育、行为干预和药物治疗等方面。通过各级卫生部门及行政单位大力宣传吸烟的危害，调动吸烟者的主观能动性积极戒烟是控烟工作取得成果的前提条件。同时对吸烟者采取有个体化的行为指导，配以必要的药物治疗，以巩固戒烟的长期成效。

除吸烟外，临床上越来越多的女性肺癌患者提示可能还有其它更多的原因参与肺癌的发生，包括女性被动吸烟、油烟暴露、精神压抑等。实际上，大气中的烟草烟尘由吸烟者呼出的主流烟尘和不经吸烟者吸收的侧流烟尘两部分组成。据报道，一支香烟燃烧所产生的主流烟中，强致癌物N-二甲基亚硝胺含量为4.1 μg-31.1 μg，而侧流烟中则达597 μg-735 μg^[2]^，因而有学者认为被动吸烟的危害大于主动吸烟。本研究中11.34%的女性有明确的被动吸烟，大部分来源于丈夫，这是对女性健康构成极大威胁的重要因素。可见，加强戒烟工作，包括家庭与公共场所的戒烟，既能降低主动吸烟者的发病危险，又可切实保护其他个体的身体健康。

此外，调查还显示15%左右的个体有长期油烟暴露史，83.05%为女性。有研究^[2]^表明吸入烹调油烟的大鼠，其血、肺及肝组织中的超氧化物歧化酶（superoxide dismutase, SOD）的活性显示明显下降，而长期接触烹调油烟的人群，血浆中的脂质过氧化物（lipid peroxides, LPO）的含量升高，Vit C、Vit E、β-胡萝卜素及红细胞中SOD以及血谷胱苷肽的含量及活性均明显降低。烹调油烟中所存在的毒性物质，可能造成基因突变、染色体损伤等，进而增加个体的肺癌发病风险。对这部分人群，除通过改善室内通风条件、保证空气流通外，加强宣传教育、普及抽油烟机等的使用也是重要手段之一。与油烟暴露相对应的是职业性粉尘、石棉、矿物等接触。5.86%的调查对象有类似职业暴露，完善职业防护措施、重视定期体格检查是保障该群体的唯一有效途径。

以上各种不良环境因素均可通过外在干预进行控制。然而，调查中另有部分个体因精神压抑、遗传易感性等原因而导致肺癌发病风险增加。精神因素可能影响机体的免疫功能，改变免疫状态，降低人体的免疫监视和免疫杀伤功能，增加肺癌发病危险。近期一项*meta*分析^[14]^提示，长期处于精神压抑状态发生肺癌的OR值为2.26（95%CI: 1.84-2.79），再次强调了关注个体精神健康的重要。此外，从20世纪60年代开始，就已有研究^[15, 16]^证实了肺癌的家族聚集现象。近来飞速发展的分子生物学技术，更为研究肺癌的遗传易感性提供了重要工具。本次调查中有525例（7.24%）居民一、二级亲属中有肿瘤家族史；141例（1.94%）有肺癌家族史，其中24例（0.33%）二者兼有。对于这部分个体，更应加强控制不良环境因素，如吸烟、被动吸烟、油烟暴露、矿物、石棉接触等的刺激；此外，易感基因筛选、定期的体格检查等也是该群体的个体化防护手段。

### 3.3 危险因素在人群中的分布

如前所述，肺癌的发生与内外环境刺激均密切相关。本次调查显示，外界环境的不良刺激仍是导致成都市40岁以上居民肺癌发生风险增加的主要原因，其中男性主要是吸烟，吸烟率达48.58%；而女性则主要为油烟暴露和被动吸烟，分别为24.41%和11.34%。在1 547例高危个体中，这种分布特点更为明显。高危男性有96.83%（1 284/1 326）吸烟；高危女性有67.87%（150/221）油烟暴露，49.32%（109/221）有被动吸烟。可见，对不同性别的个体，降低肺癌发生风险的有效干预方法有所差异。遗传易感性作为一种内源性危险因素，用常规方法不易进行调控。在本次调查中，不到10%的居民有肿瘤家族史。再次证实：个体的肺癌危险因素大部分是可控制的，其发病危险通过改变不良生活习惯、改善环境等方法可有效降低。

## 4 结论

综上所述，成都市居民的肺癌危险因素状况不容乐观，约有20%的个体属于肺癌高危人群，这包括吸烟、女性被动吸烟、油烟暴露、遗传易感性等多个因素所致。除继续大力推进控烟行动外，对于有其它危险因素的个体，应采取不同的、有针对性的措施对其进行健康教育和控制，以减少肺癌的发生危险，增进人民群众身体健康。

附表1：http://www.lungca.org/survey/2010-08-oa-0106-survey.doc
